# Complete Genome Sequence of *Herbiconiux* sp. Strain L3-i23 Isolated from Soil in Japan

**DOI:** 10.1128/mra.00815-22

**Published:** 2022-10-17

**Authors:** Miona Yajima, Yoshi Yamano, Daichi Morita, Sachiko Sugimoto, Teruo Kuroda, Katsuyoshi Matsunami

**Affiliations:** a Department of Pharmacognosy, Graduate School of Biomedical and Health Sciences, Hiroshima University, Hiroshima, Japan; b Department of Microbiology, Graduate School of Biomedical and Health Sciences, Hiroshima University, Hiroshima, Japan; University of Arizona

## Abstract

This study describes the complete genome sequence of *Herbiconiux* sp. strain L3-i23, acquired from an assembly of long reads and subsequently polished using short reads. The complete genome comprises a 3,139,863-bp chromosome with a GC content of 69.51% and a circular plasmid (39,507 bp).

## ANNOUNCEMENT

*Herbiconiux* sp. strain L3-i23 was isolated during an attempt to domesticate microorganisms that are uncultured by using a special culture device, as described below, to find sources of secondary metabolites that have never been explored before. Genome sequencing was then conducted to predict the production potential of the secondary metabolites of the obtained microorganism. The genome sequence of strain L3-i23 indicated the presumed presence of several polyketide and terpenoid biosynthesis gene clusters, making it a promising source for secondary metabolites. In this study, we present the complete genome sequence of *Herbiconiux* sp. strain L3-i23, isolated from soil of the ruins of Nissyoen in Hiroshima, Japan (34°25′N, 132°26′E), in December 2020.

The isolation and cultivation of *Herbiconiux* sp. strain L3-i23 were achieved using a culture device created in our laboratory that functions similarly to that developed by Nichols et al. (1) for culturing uncultured microorganisms. For details on the device’s size, materials, and function and the isolation and culture technique, see https://doi.org/10.6084/m9.figshare.19336745. To yield genomic DNA, strain L3-i23 was cultured at 25°C for 3 days in Waksman broth, and high-molecular-weight genomic DNA was obtained using a phenol-chloroform extraction method (2).

The short-read sequencing library was prepared using the NEBNext Ultra II FS DNA library preparation kit (New England Biolabs Japan, Tokyo, Japan), according to the manufacturer’s protocols. First, paired-end sequencing (150 bp) was conducted using a MiSeq instrument, generating 1,122,325 paired-end reads. Second, a DNA library was prepared for long-read sequencing using the rapid barcoding kit (Oxford Nanopore Technologies). The prepared library was applied to an FLO-MIN106 R9.41 flow cell (Oxford Nanopore Technologies). The long-read sequences, base-called using Guppy high-accuracy mode v.4.2.3, generated 279,136 reads with an average length of 5,834 bp during a 24-h run time. Next, the low-quality short reads were trimmed with TrimGalore v.0.6.7 (F. Krueger, TrimGalore [https://github.com/FelixKrueger/TrimGalore]), and the trimmed reads were filtered to eliminate the PhiX control sequence using BBDuk v38.96 (B. Bushnell, BBMap [https://sourceforge.net/projects/bbmap/]). The nanopore reads were first filtered using Porechop 0.2.4 (3) to eliminate sequence adapters, followed by NanoFilt v.2.7.1 (4) to eliminate low-quality reads (average *Q* value > 8.0; length > 1,000). Using Flye v2.7 (5), a long-read assembly was conducted (–genome-size 3.5-M), and it was polished with the short reads using Pilon v1.24 (6). Default parameters were used for all the software unless otherwise stated.

Genome assembly generated two contigs of 3,179,370 bp, which included a single circular chromosome (3,139,863 bp) with G+C content of 69.51% and a circular plasmid (39,507 bp). The sequencing coverage was 53× for the MiSeq data and 482× for the Nanopore data. Automatic annotation was conducted using the DFAST annotation pipeline (7). Taxonomic assignation and calculation of the average nucleotide identity value of the assembled sequence were conducted using HMMER (8) and FastANI (9) by DFAST, respectively, and the closest match was Herbiconiux flava (GenBank accession number GCA_013409865.1), with an average nucleotide identity value of 79.608%. Using DFAST, 3,039 coding sequences were predicted.

### Data availability.

The complete genome sequences were deposited at DDBJ/EMBL/GenBank under accession numbers AP025737 (genome) and AP025738 (plasmid). The raw sequencing data were deposited in the DDBJ SRA database under the accession numbers DRR336795 (MiSeq) and DRR336796 (MinION).
